# Associations of academic stress with anxiety and depressive symptoms: the mediating role of emotional eating

**DOI:** 10.3389/fnut.2026.1826577

**Published:** 2026-05-04

**Authors:** Pan Ding, Junxiu Wang

**Affiliations:** 1 School of Mental Health, Wenzhou Medical University, Wenzhou, China; 2 The Affiliated Kangning Hospital of Wenzhou Medical University, Wenzhou, China

**Keywords:** academic stress, anxiety symptoms, Chinese adolescents and young adults, depressive symptoms, emotional eating

## Abstract

**Background:**

Academic stress is a prominent stressor during adolescence. This study aimed to examine the multidimensional associations of academic stress with anxiety and depressive symptoms, as well as the potential mediating role of emotional eating.

**Methods:**

A cross-sectional survey was conducted among 608 adolescents and young adults (mean age: 18.78 years, girl: 66.61%) in southern China between July and September 2025. Academic stress was assessed using a validated multidimensional scale covering parental, self-imposed, teacher, and social pressure. Logistic regression, restricted cubic spline models, and structural equation modeling were used to evaluate associations, dose–response relationships, and potential mediation effects.

**Results:**

The prevalence of anxiety and depressive symptoms was 24.18% and 34.38%. An approximately linear dose–response association was observed between academic stress and both outcomes. Compared with participants in the lowest stress quartile, those in the highest quartile had higher odds of anxiety (OR = 8.52, 95% CI: 4.04–18.29, *P* < 0.001) and depressive symptoms (OR = 5.64, 95% CI: 3.32–9.92, *P* < 0.001). All four academic stress dimensions were independently associated with both outcomes, with social pressure showing the strongest associations. Emotional eating was identified as a potential behavior-related factor partly involved in these associations, accounting for 9.98% of the association with anxiety and 4.79% with depressive symptoms. Across stress dimensions, the indirect associations involving emotional eating were more pronounced for teacher and parental pressures.

**Conclusions:**

Academic stress was positively associated with anxiety and depressive symptoms. Emotional eating may represent a potential behavior-related correlate involved in these associations.

## 1 Introduction

Adolescence and young adulthood are critical developmental periods characterized by rapid biological, psychological, and social changes, during which individuals are particularly vulnerable to mental health problems (1, 2). Anxiety and depressive disorders are among the most prevalent mental health conditions in adolescents and young adults (3) and represent major contributors to disease burden, impaired academic functioning, and adverse long-term health outcomes. Epidemiological studies indicated that nearly 15–20% of children and adolescents experience an anxiety disorder during their lifetime (4), while the prevalence of depressive symptoms increases from childhood to adolescence, with nearly 20% of adolescents and young adults experiencing a depressive episode by late adolescence (5). Identifying modifiable psychosocial risk factors and behavioral pathways underlying adolescent mental health problems is therefore a public health priority.

Academic stress encompasses a range of pressures related to academic performance, expectations from parents and teachers, peer comparison, and self-imposed standards (6). Academic stress has increasingly been recognized as a salient stressor among Chinese adolescents and young adults (7), where educational systems are highly performance-oriented and academic achievement is closely tied to family expectations and future socioeconomic opportunities (8). A growing body of research has demonstrated that elevated academic stress is associated with increased risks of anxiety and depressive symptoms among adolescents and young adults (7–9). However, much of the existing literature has conceptualized academic stress as a unidimensional construct, limiting understanding of how specific stress domains differentially contribute to mental health outcomes.

Beyond direct psychological effects, academic stress may influence adolescent mental health through behavioral pathways. Emotional eating—eating in response to negative emotions rather than physiological hunger (10)—has been linked to stress exposure and impaired emotion regulation (11). The relationships among academic stress, emotional eating, and mental health can be understood through stress and coping theory (12) and emotion regulation models (13). According to stress and coping theory, when adaptive coping resources are insufficient, adolescents and young adults may be more likely to rely on maladaptive patterns such as emotional eating (14). Emotion regulation models similarly suggest that emotional eating may reflect a maladaptive tendency to regulate negative affect through eating rather than addressing the source of stress (15). Although this may provide temporary relief, it often fails to resolve underlying stress and may exacerbate negative emotions over time (16–18). In this context, emotional eating may be involved as a behavioral characteristic in the observed associations between academic stress and anxiety/depressive symptoms. Although prior research has linked academic stress to adolescent mental health and has also associated stress with emotional eating, relatively few studies have examined emotional eating as a potential factor involved in the associations between multidimensional academic stress and anxiety and depressive symptoms. Given that socially evaluative stressors, such as peer comparison and fear of failure (19, 20), are particularly salient during adolescence, it is plausible that these stress dimensions may be more strongly linked to emotion-driven eating behaviors and subsequent psychological distress. Clarifying these pathways may provide important insights for targeted intervention strategies.

Based on stress and coping theory and emotion regulation models, we propose that emotional eating may represent a behavior-related factor involved in the observed associations between academic stress and adverse mental health outcomes. Accordingly, using a convenience sample of offspring of medical staff and after adjusting for potential confounders (including demographic characteristics and lifestyle behaviors), we formulated the following hypotheses: Hypothesis 1: Academic stress is positively associated with anxiety and depressive symptoms, with stronger effects expected for socially evaluative stressors (e.g., parental, teacher, and social pressure) compared with self-imposed pressure; Hypothesis 2: Emotional eating partially mediates the associations between academic stress and anxiety/depressive symptoms.

## 2 Methods

### 2.1 Study design and participants

This cross-sectional survey was conducted between July and September 2025 at several general hospitals in southern China. The questionnaire was designed by experts in epidemiology, psychology, and sociology, drawing on instruments used in previous large-scale cohort studies (21, 22). It collected information on basic demographic characteristics, academic stress, lifestyle behaviors, and health-related factors. Using a convenience sampling method, we recruited school-age offspring of medical staff as participants. Prior to individual face-to-face interviews, researchers provided a detailed explanation of the study's background, objectives, and confidentiality measures. Participants were explicitly informed of their right to withdraw from the study at any time without penalty, and were assured that refusal to participate or incomplete submission would entail no adverse consequences. Written informed consent was obtained from all participants, and from parents or legal guardians for participants aged under 18 years. This study was conducted in accordance with the Declaration of Helsinki and approved by the Institutional Research Ethics Committee of Wenzhou Medical University (YJ-2024-09-01).

We recruited 608 adolescents and young adults aged between 9 and 25 years, all of whom completed the questionnaire in full (100% response rate). They constituted the final sample for this study.

### 2.2 Assessment of academic stress

Academic stress was assessed using the 21-item academic stress scale (23, 24), which comprises four dimensions: parental pressure (6 items), self-imposed pressure (6 items), teacher pressure (5 items), and social pressure (4 items) (Supplementary Table S1). Each item is rated on a 5-point Likert scale ranging from 1 (“strongly disagree”) to 5 (“strongly agree”). Total scores range from 21 to 105, with higher scores indicating greater levels of academic stress. The academic stress scale has demonstrated satisfactory validity and reliability in previous studies among Chinese adolescents and young adults (25). In this study, the scale exhibited excellent internal consistency, with a Cronbach's alpha coefficient of 0.936. Academic stress was subsequently categorized into four groups based on the median and quartiles for further analysis.

### 2.3 Assessment of anxiety symptoms

Anxiety symptoms were assessed using the Generalized Anxiety Disorder 7-item scale (GAD-7)(26), which evaluates the frequency of anxiety-related symptoms over the past 2 weeks, including nervousness, inability to control worry, excessive worry, difficulty relaxing, restlessness, irritability, and fear that something awful might happen. Each item is rated on a four-point Likert scale ranging from 0 (“not at all”) to 3 (“nearly every day”), yielding a total score ranging from 0 to 21, with higher scores indicating greater severity of anxiety symptoms (27). In the present study, the GAD-7 demonstrated excellent internal consistency, with a Cronbach's alpha coefficient of 0.962. Consistent with established classification criteria in prior studies, a GAD-7 score of ≥ 7 was used to define the presence of anxiety symptoms (28). The GAD-7 has also been validated as an effective and efficient instrument for assessing anxiety symptoms among Chinese adolescents and young adults (29).

### 2.4 Assessment of depressive symptoms

Depressive symptoms were assessed using the 2-item Patient Health Questionnaire (PHQ-2) (30), which measures the frequency of core depressive symptoms during the past 2 weeks, including fatigue or low energy and feelings of low mood, depression, or hopelessness. Each item is rated on the same four-point Likert scale (0 = “not at all” to 3 = “nearly every day”), resulting in a total score ranging from 0 to 6, with higher scores reflecting more severe depressive symptoms (31). Compared with the PHQ-9, the PHQ-2 demonstrates comparable sensitivity and specificity and has therefore been widely used in epidemiological studies (32). Moreover, previous studies have shown that the PHQ-2 possesses robust predictive validity for assessing depressive symptoms among adolescents and young adults across diverse cultural and national contexts (31, 33). Following commonly used cutoff criteria in prior research, a PHQ-2 score of ≥ 2 was used to indicate the presence of depressive symptoms (33, 34).

### 2.5 Assessment of emotional eating

Emotional eating was assessed using the emotional eating dimension of the Dutch Eating Behavior Questionnaire (DEBQ) (35). This 9-item subscale measures the tendency to eat in response to negative emotional states over the past year, including anger, frustration, upset, loneliness, disappointment, nervousness, anticipation of unpleasant events, boredom, and fear. Each item was rated on a 5-point Likert scale ranging from 1 (“never”) to 5 (“always”), with higher scores indicating a greater propensity toward emotional eating (36). The Chinese version of the emotional eating questionnaire has been validated in Chinese adolescents and young adults, demonstrating excellent internal consistency (Cronbach's alpha: 0.964), high test–retest reliability (*r* = 0.854), and good structural and criterion validity (37). In the present study, the selected items showed excellent internal consistency, with a Cronbach's alpha coefficient of 0.956. Accordingly, emotional eating, as assessed in this study, was treated as a behavior-related tendency reflecting eating in response to negative emotions over the past year. Given the cross-sectional design, it was interpreted as a correlate statistically involved in the observed associations rather than as a temporally ordered mediator (38).

### 2.6 Assessment of covariates

Based on previous research (39, 40), a range of covariates was included to control for potential confounding effects, encompassing general demographic characteristics and lifestyle behaviors. General demographic variables included sex (boy or girl), age (continuous), place of residence (city, town, or rural), educational level (primary school, junior high school, senior high school, or university), and family economic status (good or poor). Lifestyle-related variables comprised sleep duration (continuous), sleep quality (good or poor), social media (continuous), sedentary (continuous), physical activity (continuous), dietary diversity (continuous), BMI (continuous), and body image (continuous).

Dietary diversity was assessed using a 10-item food frequency questionnaire (FFQ) (21) covering major food groups, including meat, aquatic products and seafood, fresh vegetables, fruits, eggs, soy products, milk and dairy products, preserved vegetables, high-sugar foods, and fried foods. Responses were rated on a 4-point Likert scale: 1 (“rarely or never”), 2 (“1–2 times per week”), 3 (“3–5 times per week”), and 4 (“daily”). Preserved vegetables, high-sugar foods, and fried foods were reverse-coded. The dietary diversity score ranges from 10 to 40, with higher scores indicating a more balanced dietary pattern (41). Body mass index (BMI) was calculated as weight in kilograms divided by height in meters squared (kg/m^2^) (42). Body image was assessed using a 6-item scale (43) evaluating satisfaction with overall appearance, body shape, posture, body weight, and perceived physical attractiveness. Each item was rated on a 9-point Likert scale ranging from 1 (“extremely dissatisfied”) to 9 (“extremely satisfied”). Item scores were summed to yield a total body image score ranging from 6 to 54, with higher scores indicating greater body image satisfaction (44).

### 2.7 Statistical analyses

To compare the basic characteristics with different levels of academic stress, the ANOVA was employed for normally distributed continuous data, the Kruskal-Wallis test for ordinal data, and the Chi-square test for unordered categorical data. Logistic regression models were used to analyze the independent associations, and the results were described using odds ratios (*ORs*) and 95% confidence intervals (*CIs*). The crude model was unadjusted. The adjusted model controlled for potential confounders including sex, age, place of residence, education, family economic status, sleep duration, sleep quality, social media, sedentary, physical activity, dietary diversity, BMI, and body image. *P* for trend was obtained by analyzing the academic stress variable as a continuous variable. Additionally, we used restricted cubic splines to assess linearity and trends in the dose-response associations of academic stress with anxiety and depressive symptoms.

To further validate the consistency of the results, stratified analyses were conducted for sex, age, education, BMI, sleep duration, social media, physical activity, and sedentary. The *P* for interaction was obtained by comparing the likelihood ratios of the terms with and without the multiplicative interaction. To verify the robustness of the associations, several sensitivity analyses were conducted. Firstly, to minimize potential confounding by environmental factors, we restricted participants to those with good sleep quality, those residing in towns or cities, and those with good family economic status. Secondly, we reassessed the associations after excluding participants aged younger than 10 years or those with a BMI > 24 kg/m^2^ to reduce the impact of extreme age or weight status. Finally, to account for selection bias and potential confounding, we applied inverse probability weighting and reanalyzed the associations between academic stress and mental health outcomes.

Furthermore, we examined whether emotional eating was statistically involved as a potential mediator in the associations between academic stress (and its four dimensions) and anxiety/depressive symptoms using structural equation modeling (SEM). Given the cross-sectional design, these analyses were interpreted as reflecting associational rather than temporal or causal mediation. The latent construct of academic stress was specified using its four observed dimensions. Satisfactory model fit indices included Comparative Fit Index (CFI) ≥ 0.90, Tucker-Lewis Index (TLI) ≥ 0.90, Root Mean Square Error of Approximation (RMSEA) ≤ 0.08, and Standardized Root Mean Square Residual (SRMR) ≤ 0.08(45). The direct and indirect effects were estimated, and the proportion mediated was calculated to quantify the magnitude of the mediation.

All statistical analyses were performed using SPSS version 26.0 and R software version 4.5.2 (http://www.R-project.org). A two-sided *P* < 0.05 was considered statistically significant.

## 3 Results

### 3.1 Basic characteristics of the study population

A total of 608 participants (mean age = 18.78 years; 66.61% girl) were enrolled in this study. The majority of participants resided in cities (50.82%), with 78.94% having attained an education level of senior high school or above, and 60.03% reporting good family economic status.

After grouping by quartiles of academic stress, compared with participants in the lower academic stress group (Q1/Q2), those in the higher academic stress group (Q3/Q4) had a higher proportion of boys, poor family economic status, and poor sleep quality. Additionally, participants with higher academic stress showed longer sedentary time, lower dietary diversity scores, higher emotional eating scores, and poorer body image (all *P* < 0.05; Table 1).

**Table 1:** Basic characteristics of participants by academic stress (*n* = 608).

Variables	Academic stress					*P*-value
	Total (*n* = 608)	Q1 (*n* = 160)	Q2 (*n* = 149)	Q3 (*n* = 139)	Q4 (*n* = 160)	
Age, years, Mean ± SD	18.78 ± 3.25	18.46 ± 3.59	19.05 ± 3.05	18.43 ± 3.38	19.18 ± 2.90	0.155
Sex, *n* (%)						**0.026**
Boy	203 (33.39)	55 (34.38)	36 (24.16)	48 (34.53)	64 (40.00)	
Girl	405 (66.61)	105 (65.62)	113 (75.84)	91 (65.47)	96 (60.00)	
Place of residence, *n* (%)						0.714
City	309 (50.82)	86 (53.75)	83 (55.70)	68 (48.92)	72 (45.00)	
Town	156 (25.66)	36 (22.50)	38 (25.50)	37 (26.62)	45 (28.12)	
Rural	143 (23.52)	38 (23.75)	28 (18.79)	34 (24.46)	43 (26.88)	
Education, *n* (%)						0.662
Primary school	93 (15.30)	33 (20.62)	17 (11.41)	25 (17.99)	18 (11.25)	
Junior high school	35 (5.76)	7 (4.38)	8 (5.37)	10 (7.19)	10 (6.25)	
Senior high school	439 (72.20)	109 (68.12)	113 (75.84)	98 (70.50)	119 (74.38)	
University	41 (6.74)	11 (6.88)	11 (7.38)	6 (4.32)	13 (8.12)	
Family economic, *n* (%)						**<** **0.001**
Good	365 (60.03)	114 (71.25)	93 (62.42)	76 (54.68)	82 (51.25)	
Poor	243 (39.97)	46 (28.75)	56 (37.58)	63 (45.32)	78 (48.75)	
Sleep duration, h, Mean ± SD	8.95 ± 2.14	9.13 ± 2.05	9.00 ± 2.04	8.90 ± 2.21	8.75 ± 2.25	0.777
Sleep quality, *n* (%)						**0.005**
Good	561 (92.27)	156 (97.50)	141 (94.63)	125 (89.93)	139 (86.88)	
Poor	47 (7.73)	4 (2.50)	8 (5.37)	14 (10.07)	21 (13.12)	
Social media, h, Mean ± SD	5.25 ± 3.94	4.94 ± 4.38	5.30 ± 3.43	5.15 ± 3.83	5.58 ± 4.03	0.659
Sedentary, h, Mean ± SD	5.10 ± 4.66	4.28 ± 3.53	4.92 ± 3.04	5.85 ± 5.25	5.44 ± 6.04	**0.036**
Physical activity, h, Mean ± SD	1.24 ± 1.52	1.18 ± 1.33	1.38 ± 1.94	1.23 ± 1.58	1.18 ± 1.15	0.730
Dietary diversity, Mean ± SD	29.46 ± 3.94	30.31 ± 4.13	29.78 ± 3.42	29.42 ± 3.70	28.36 ± 4.18	**<** **0.001**
Emotional eating, Mean ± SD	20.82 ± 8.88	17.07 ± 8.11	20.41 ± 7.50	20.98 ± 8.55	24.82 ± 9.45	**<** **0.001**
BMI, kg/m^2^, Mean ± SD	20.85 ± 3.75	20.43 ± 3.89	21.33 ± 3.67	20.55 ± 3.23	21.08 ± 4.07	0.113
Body image, Mean ± SD	34.88 ± 9.63	38.59 ± 9.89	34.60 ± 8.67	32.98 ± 8.23	33.08 ± 10.35	**<** **0.001**

BMI, Body Mass Index; SD, Standard Deviation.

Academic stress was divided into four groups using quartiles (Q1 < 44, Q2 < 53, Q3 < 63, Q4 ≥ 63).

ANOVA was employed to compare normally distributed data, the chi-square test was used for the comparison of unordered categorical data, and the Kruskal-Wallis test was applied to analyze ordinal data. Bold values indicated statistical significance *P* < 0.05.

### 3.2 Association between academic stress and anxiety/depressive symptoms

As presented in Table 2, in the crude model, higher levels of academic stress were significantly associated with higher odds of both anxiety and depressive symptoms. Compared with participants in the lowest quartile of academic stress (Q1), those in the second (Q2), third (Q3), and highest quartile (Q4) had higher odds of anxiety symptoms in the crude model (Q2: 3.06, 95% CI: 1.37–6.86; Q3: 5.86, 95% CI: 2.71–12.69; Q4: 16.36, 95% CI: 7.80–34.31; *P* for trend < 0.001). A similar pattern was observed for depressive symptoms, with higher *ORs* in Q2 (2.40, 95% CI: 1.32–4.35), Q3 (5.64, 95% CI: 3.17–10.02), and Q4 (8.77, 95% CI: 5.00–15.41) compared with Q1 (*P* for trend < 0.001). After adjustment for potential confounders, the associations were attenuated. Participants in the higher academic stress groups continued to have higher odds of anxiety symptoms (Q3: OR = 3.83, 95% CI: 1.85–8.28; Q4: OR = 8.52, 95% CI: 4.04–18.29; *P* for trend < 0.001). A similar pattern was observed for depressive symptoms, with adjusted *ORs* of 3.91 (95% CI: 2.27–6.94) for Q3 and 5.64 (95% CI: 3.32–9.92) for Q4 compared with the lowest stress group (*P* for trend < 0.001).

**Table 2:** Association of academic stress with anxiety/depressive symptoms (*n* = 608).

Health outcomes	*n* (%)	Academic stress, *OR* (95%*CI*)				*P* for trend
		Q1 (*n* = 160)	Q2 (*n* = 149)	Q3 (*n* = 139)	Q4 (*n* = 160)	
Crude model						
Anxiety symptoms	147 (24.18)	1.00 (Ref.)	**3.06 (1.37–6.86)**	**5.86 (2.71–12.69)**	**16.36 (7.80–34.31)**	**<** **0.001**
Depressive symptoms	209 (34.38)	1.00 (Ref.)	**2.40 (1.32–4.35)**	**5.64 (3.17–10.02)**	**8.77 (5.00–15.41)**	**<** **0.001**
Adjusted model ^a^						
Anxiety symptoms	147 (24.18)	1.00 (Ref.)	2.26 (0.98–5.20)	**3.83 (1.85–8.28)**	**8.52 (4.04–18.29)**	**<** **0.001**
Depressive symptoms	209 (34.38)	1.00 (Ref.)	1.75 (0.96–3.25)	**3.91 (2.27–6.94)**	**5.64 (3.32–9.92)**	**<** **0.001**

OR, Odds Ratio; CI, Confidence Interval.

Academic stress was divided into four groups using quartiles (Q1 < 44, Q2 < 53, Q3 < 63, Q4 ≥ 63).

^**a**^ Adjusted for sex (boy, girl), age (continuous), place of residence (city, town, rural), education (primary school, junior high school, senior high school, university), family economic status (good, poor), sleep duration (continuous), sleep quality (good, poor), social media (continuous), sedentary (continuous), physical activity (continuous), dietary diversity (continuous), BMI (continuous), and body image (continuous).

Bold values indicated statistical significance *P* < 0.05.

**Figure 1:**
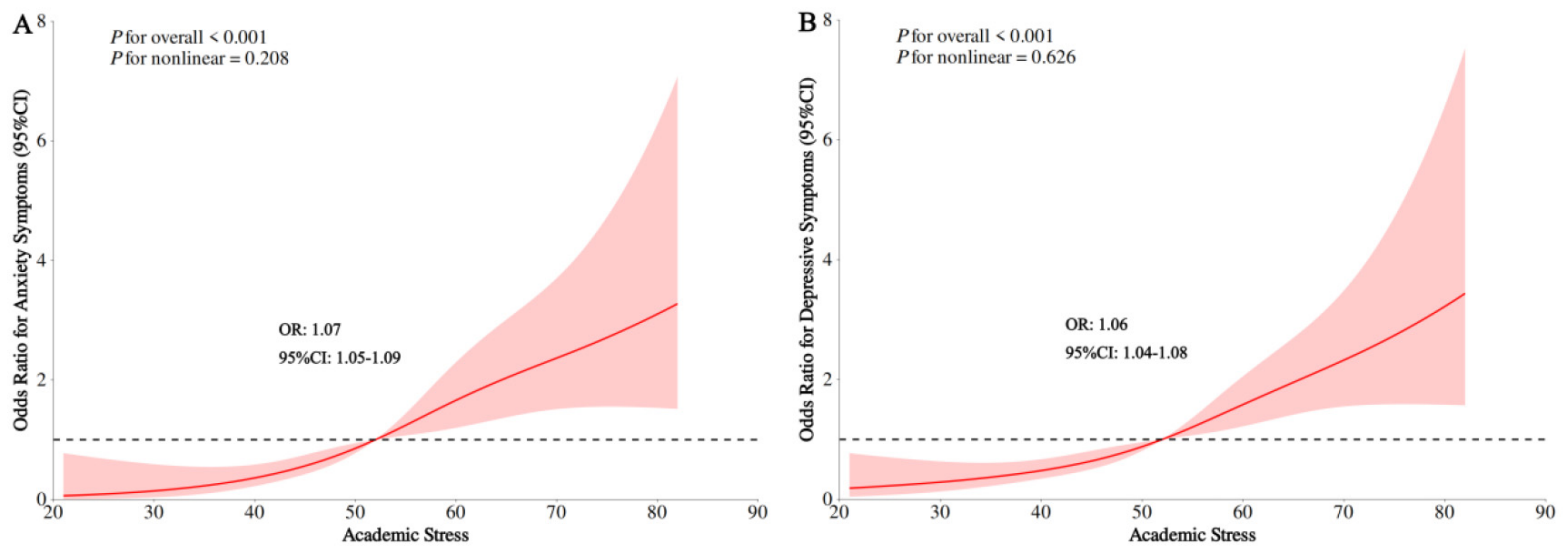
Restricted cubic spline for the association between academic stress and anxiety/ depressive symptoms. **(A)** Academic stress with anxiety symptoms, **(B)** Academic stress with depressive symptoms. Adjusted for sex (boy, girl), age (continuous), place of residence (city, town, rural), education (primary school, junior high school, senior high school, university), family economic status (good, poor), sleep duration (continuous), sleep quality (good, poor), social media (continuous), sedentary (continuous), physical activity (continuous), dietary diversity (continuous), BMI (continuous), and body image (continuous).

Restricted cubic spline analyses showed significant positive associations between academic stress and both anxiety (OR = 1.07, 95% CI: 1.05–1.09, *P* < 0.001) and depressive symptoms (OR = 1.06, 95% CI: 1.04–1.08, *P* < 0.001). The overall associations were statistically significant (all *P* for overall < 0.001), with no indication of non-linearity for either anxiety (*P* for non-linearity = 0.208; Figure 1A) or depressive symptoms (*P* for non-linearity = 0.626; Figure 1B), suggesting approximately linear associations across the observed range of academic stress.

### 3.3 Stratification and sensitivity analysis

**Figure 2:**
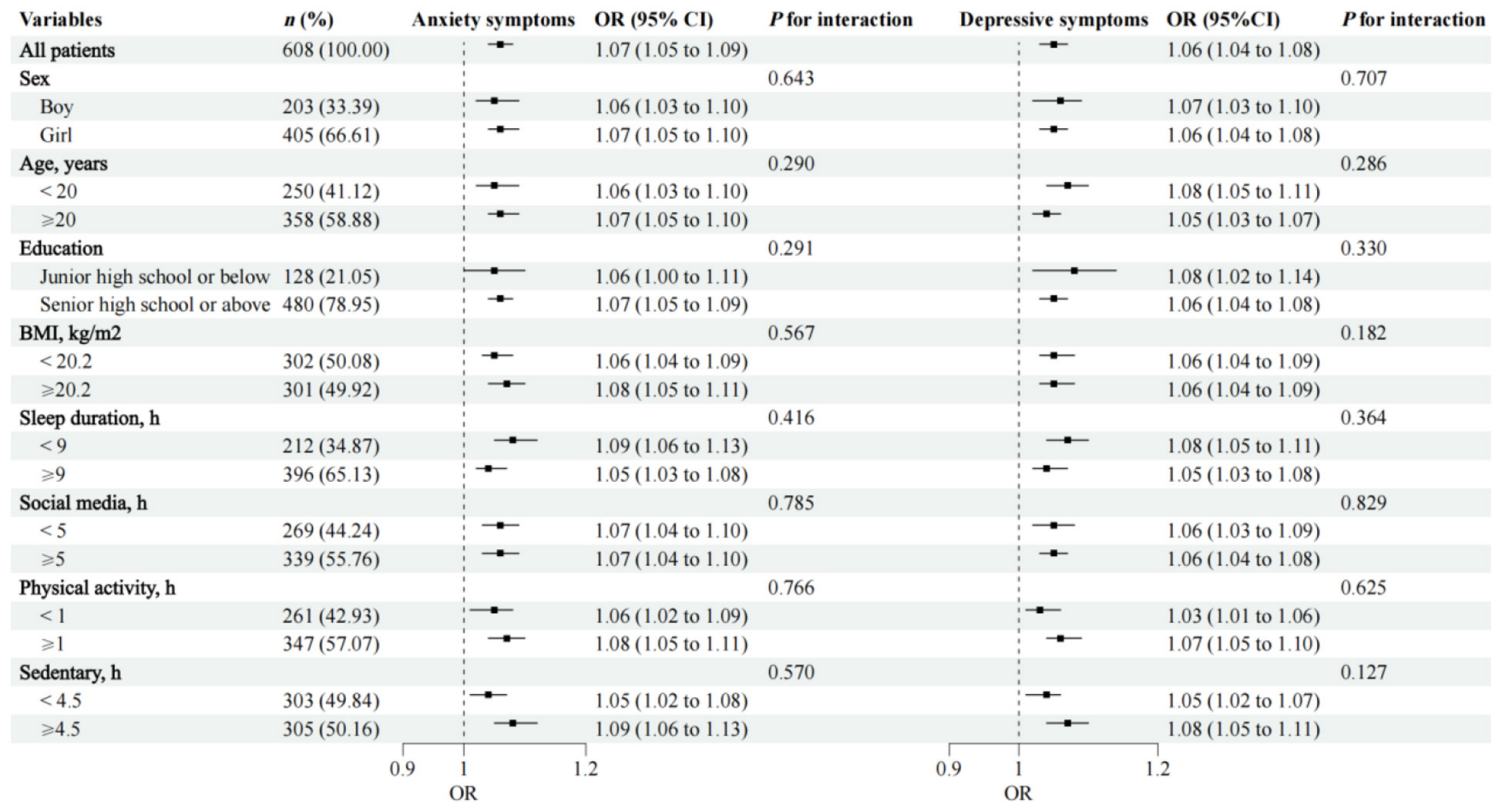
Association of academic stress with anxiety/depressive symptoms, stratification analyses. Age, BMI, sleep duration, social media, physical activity, and sedentary were grouped according to median. Adjusted for sex (boy, girl), age (continuous), place of residence (city, town, rural), education (primary school, junior high school, senior high school, university), family economic status (good, poor), sleep duration (continuous), sleep quality (good, poor), social media (continuous), sedentary (continuous), physical activity (continuous), dietary diversity (continuous), BMI (continuous), and body image (continuous). Except for the factor itself.

In stratified analyses, the associations between academic stress and both anxiety and depressive symptoms were generally consistent across subgroups of sex, age, education, BMI, sleep duration, social media, physical activity, and sedentary, with all *P* for interaction > 0.05 (Figure 2).

In sensitivity analyses, the observed associations remained materially unchanged when analyses were restricted to participants with good sleep quality, those residing in towns or cities, and those with good family economic status. Similar results were observed after excluding participants younger than 10 years or those with a BMI greater than 24 kg/m^2^. In addition, after applying inverse probability weighting, the associations between academic stress and both outcomes remained statistically significant (Supplementary Table S2).

### 3.4 Associations of academic stress dimensions with anxiety/depressive symptoms

We further examined the associations of the four academic stress dimensions (parental pressure, self-imposed pressure, teacher pressure, and social pressure) with both outcomes after multivariable adjustment. All four dimensions were positively associated with both outcomes (all *P* < 0.001), with no evidence of non-linearity (all *P* > 0.05; Supplementary Figures S1, S2). As shown in Table 3, for anxiety symptoms, the strongest association was observed for social pressure (OR = 1.44), followed by parental pressure (OR = 1.17), teacher pressure (OR = 1.17), and self-imposed pressure (OR = 1.15) (all *P* < 0.001). Similarly, for depressive symptoms, social pressure showed the strongest association (OR = 1.39), whereas self-imposed pressure, teacher pressure, and parental pressure showed relatively smaller but still significant associations (OR = 1.16, 1.15, and 1.11, respectively; all *P* < 0.001).

**Table 3:** Associations between dimensions of academic stress and anxiety/depressive symptoms.

Dimensions of academic stress	Mean ±SD	*OR* for anxiety symptoms (95% *CI*)	*P*-value
Parental pressure	14.53 ± 4.92	**1.17 (1.11–1.22)**	**<** **0.001**
Self-imposed pressure	15.91 ± 5.23	**1.15 (1.10–1.21)**	**<** **0.001**
Teacher pressure	11.47 ± 3.80	**1.17 (1.10–1.24)**	**<** **0.001**
Social pressure	10.02 ± 2.60	**1.44 (1.30–1.58)**	**<** **0.001**
Dimensions of academic stress	Mean ±SD	*OR* for depressive symptoms (95% *CI*)	*P*-value
Parental pressure	14.53 ± 4.92	**1.11 (1.07–1.16)**	**<** **0.001**
Self-imposed pressure	15.91 ± 5.23	**1.16 (1.11–1.22)**	**<** **0.001**
Teacher pressure	11.47 ± 3.80	**1.15 (1.09–1.22)**	**<** **0.001**
Social pressure	10.02 ± 2.60	**1.39 (1.27–1.52)**	**<** **0.001**

Adjusted for sex (boy, girl), age (continuous), place of residence (city, town, rural), education (primary school, junior high school, senior high school, university), family economic status (good, poor), sleep duration (continuous), sleep quality (good, poor), social media (continuous), sedentary (continuous), physical activity (continuous), dietary diversity (continuous), BMI (continuous), and body image (continuous).

Bold values indicated statistical significance *P* < 0.05.

### 3.5 Emotional eating in the association between academic stress and anxiety/depressive symptoms

**Figure 3:**
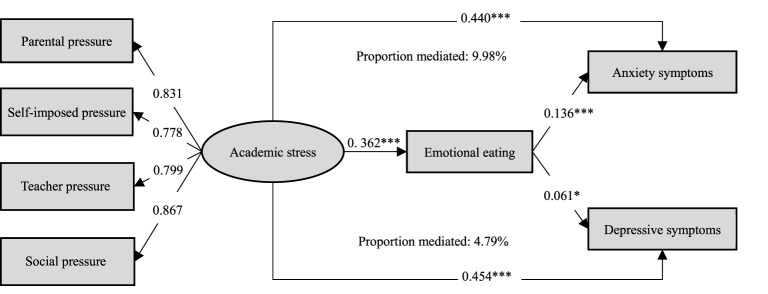
Mediating pathway effects of emotional eating on the association between academic stress and anxiety/depressive symptoms. *P* < 0.05*, *P* < 0.001***.

Structural equation modeling was conducted to evaluate the hypothesized model (Figure 3), which showed satisfactory fit to the data (CFI = 0.990, TLI = 0.980, SRMR = 0.023, RMSEA = 0.059). Academic stress was specified as a latent construct indicated by parental pressure (β = 0.831), self-imposed pressure (β = 0.778), teacher pressure (β = 0.799), and social pressure (β = 0.867). Within the SEM framework, academic stress was positively associated with emotional eating (β = 0.362, *P* < 0.001), and emotional eating was positively associated with anxiety symptoms (β = 0.136, *P* < 0.001) and depressive symptoms (β = 0.061, *P* < 0.05). Academic stress was also statistically associated with anxiety symptoms (β = 0.440, *P* < 0.001) and depressive symptoms (β = 0.454, *P* < 0.001). Based on the model estimates, the proportion of the association involving emotional eating was 9.98% for anxiety symptoms and 4.79% for depressive symptoms.

**Figure 4:**
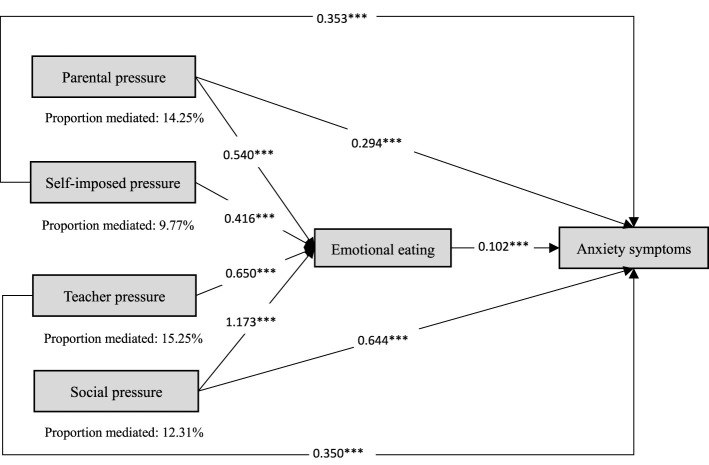
Mediating pathway effects of emotional eating on the association between dimensions of academic stress and anxiety symptoms. *P* < 0.001***.

**Figure 5:**
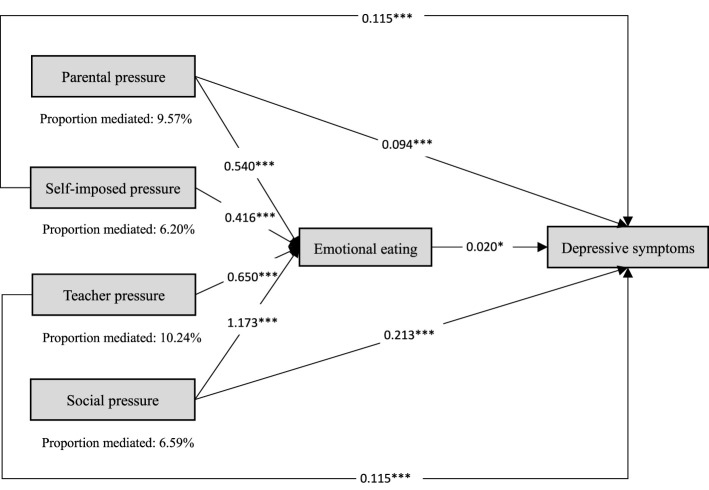
Mediating pathway effects of emotional eating on the association between dimensions of academic stress and depressive symptoms. *P* < 0.05*, *P* < 0.001***.

Further analyses by academic stress dimension showed that the proportion of the association involving emotional eating was relatively larger for teacher pressure and parental pressure than for the other dimensions. For anxiety symptoms (Figure 4), the corresponding proportions were 15.25% for teacher pressure, 14.25% for parental pressure, 12.31% for social pressure, and 9.77% for self-imposed pressure. For depressive symptoms (Figure 5), the corresponding proportions were 10.24% for teacher pressure, 9.57% for parental pressure, 6.59% for social pressure, and 6.20% for self-imposed pressure.

## 4 Discussion

### 4.1 Main findings

This cross-sectional study showed an approximately linear association between academic stress and both anxiety (OR = 1.07) and depressive symptoms (OR = 1.06) among Chinese adolescents and young adults. Compared with those experiencing low academic stress (Q1), participants with higher levels of academic stress (Q3 and Q4) had higher odds of anxiety symptoms (OR = 3.83 and 8.52, respectively) and depressive symptoms (OR = 3.91 and 5.64, respectively). All four dimensions of academic stress were independently associated with anxiety and depressive symptoms, with social pressure showing relatively stronger associations. The magnitude of these ORs, particularly in the highest academic stress group, suggests that the observed associations were not only statistically significant but also meaningful in size. In addition, emotional eating was identified as a potential behavior-related factor partly involved in the observed associations of academic stress with anxiety and depressive symptoms, accounting for 9.98% and 4.79% of the observed associations, respectively. Although these proportions were relatively modest, they may still be meaningful, as emotional eating likely represents only one of several behavioral and psychological characteristics involved in the observed associations between academic stress and adolescent mental health. Further analyses across academic stress dimensions showed that the proportion involving emotional eating was relatively larger for teacher pressure and parental pressure than for the other dimensions for both outcomes.

### 4.2 Comparison with other studies

Previous epidemiological surveys have consistently highlighted a concerning prevalence of psychological distress among university students. For instance, studies from Portugal (46), Egypt (47), and Malaysia (48) have reported high rates of anxiety (63%−75%) and depression (61%−65%). Similarly, a longitudinal study in China observed that the prevalence of anxiety (38.9%−45.8%) and depression (32.4%−37.1%) remained at elevated levels among undergraduates (49). In contrast to these findings, the present study observed a notably lower prevalence of anxiety (24.18%) and depressive symptoms (34.38%). This discrepancy may be attributed to the timing of our data collection, which occurred between July and September (summer vacation), a period likely characterized by reduced immediate academic demands. Furthermore, our sample comprised offspring of hospital staff, a group that may have had relatively better family economic conditions (60.03% reported good status) and higher health literacy than the general adolescent population, which may have been associated with a lower likelihood of severe psychological symptoms.

The findings of this study indicate that academic stress is associated with adverse mental health outcomes. Previous studies conducted among adolescents and young adults across different countries, including China (50), Spain (51), Turkey (52), and the Philippines (53), have consistently reported moderate positive associations between academic stress and both anxiety (β = 0.13–0.49) and depressive symptoms (β = 0.11–0.36). The similarity of these effect sizes across diverse cultural and educational contexts suggests that the detrimental impact of academic stress on mental health is robust. Consistent with these correlational findings, the clear and approximately linear dose–response relationships observed in our study further suggest that increasing levels of academic stress were associated with progressively poorer psychological wellbeing, rather than becoming relevant only at extreme levels (54).

Previous studies suggested that the associations of academic stress with mental health may vary across specific stress dimensions. Among adolescents and young adults in India (55), the strongest associations with anxiety were observed for teacher–student relationships (β = 0.52), fear of failure (β = 0.48), and personal inadequacy (β = 0.46). These dimensions primarily reflect performance-related concerns and perceived academic competence, suggesting that anxiety may be particularly closely related to stressors involving evaluation, expectations, and academic self-worth. Consistent with this dimensional perspective, our study further showed that social pressure and self-imposed pressure were most strongly associated with both anxiety and depressive symptoms. This pattern suggests that stressors involving social comparison and internalized academic expectations may be particularly relevant, highlighting the importance of examining specific dimensions of academic stress rather than treating it as a unitary construct.

Our analyses suggested that emotional eating might be involved as a potential behavior-related correlate in the observed associations between academic stress and adolescent mental health outcomes. First, academic stress was positively associated with emotional eating, consistent with prior research (56), suggesting that adolescents and young adults experiencing heightened academic demands may be more likely to report emotional eating tendencies (57). Second, emotional eating was positively associated with both outcomes. Rather than providing sustained emotional relief, emotional eating has been linked to impaired emotion regulation and heightened negative affect (10, 58). In our cross-sectional analyses, emotional eating was statistically consistent with partial involvement in the associations between academic stress and both outcomes, with a relatively larger proportion observed for anxiety. This pattern was consistent with the possibility that emotional eating might be more closely related to high-arousal stress responses characteristic of anxiety (59, 60), whereas its association with depressive symptoms may be comparatively weaker. Moreover, this pattern was observed across all four dimensions of academic stress, with relatively larger indirect associations for teacher and parental pressures. These findings support the relevance of emotional eating as a plausible correlate within the observed associations between academic stress and adolescent psychological distress.

### 4.3 Potential mechanisms

Several potential mechanisms may help explain the observed associations between academic stress, emotional eating, and anxiety/depressive symptoms among adolescents and young adults. First, from a stress and coping perspective, sustained academic demands may be associated with limited adaptive coping resources, which in turn may relate to greater reliance on maladaptive emotion regulation strategies such as emotional eating (61). Food intake, particularly palatable or energy-dense foods, may provide short-term emotional relief through reward-related neural pathways (62); however, such relief may be transient and may also be associated with heightened negative affect and psychological vulnerability (63). Second, chronic academic stress may be related to neuroendocrine stress responses (64, 65), including dysregulation of the hypothalamic–pituitary–adrenal (HPA) axis and elevated cortisol secretion, which have been associated with enhanced appetite and cravings for comfort foods (66). Such physiological responses may help explain why stress exposure is associated with emotion-driven eating behaviors (67), which may in turn be linked to anxiety and depressive symptoms. Third, socially evaluative stressors (68)—such as teacher pressure, parental expectations, and peer comparison—may heighten adolescents and young adults' sensitivity to failure and social judgment. These stressors can intensify self-focused negative emotions and internalized stress (69), which may increase the likelihood of eating as a compensatory coping behavior. Repeated engagement in emotional eating may also be associated with feelings of loss of control, guilt, and self-criticism (70), which may accompany more severe internalizing symptoms.

### 4.4 Strengths and limitations

Our study conceptualized academic stress as a multidimensional construct and examined emotional eating as a potential behavior-related correlate within these associations, providing a more comprehensive understanding of the relationships between academic stress and adolescent mental health outcomes. In addition, validated measurement instruments with high internal consistency and extensive covariate adjustment enhanced the robustness of the findings.

Several limitations should be carefully considered. First, the cross-sectional design limits causal interpretation, and the temporal relationships among academic stress, emotional eating, and anxiety or depressive symptoms cannot be determined. Reverse causality is possible, whereby existing mental health symptoms may influence stress perception or eating behaviors. Longitudinal studies are needed to verify the temporal sequence and further clarify these observed associations. Second, all data were self-reported, which may be subject to recall bias and social desirability bias, potentially leading to measurement error. Third, although a broad range of sociodemographic, lifestyle, dietary, and psychosocial covariates was included, residual confounding from unmeasured factors, such as personality traits or family mental health history, cannot be excluded. Fourth, the study used a convenience sample of offspring of medical staff, which may have introduced selection bias and limited generalizability, as these participants may have differed from the general adolescent population in socioeconomic conditions, health literacy, family support, and access to health-related resources. Fifth, the age range of 9–25 years spans late childhood, adolescence, and young adulthood, which may have introduced developmental heterogeneity. Although age-stratified analyses (< 20 and ≥20 years) showed no significant interaction and sensitivity analyses restricted to participants aged ≥10 years yielded similar results, this heterogeneity may still have influenced the observed associations. Finally, depressive symptoms were assessed using the PHQ-2, which, although practical and widely used for screening, includes only two core items and may not capture the full range or severity of depressive symptomatology. This may have limited the depth and precision of depression measurement in the present study.

## 5 Conclusions

This study showed that academic stress was significantly and consistently associated with anxiety and depressive symptoms among adolescents and young adults. Emotional eating was identified as a potential behavior-related factor partly involved in these associations. Notably, socially evaluative academic stressors showed relatively stronger associations with mental health outcomes. These findings highlight the potential relevance of addressing both academic stress and stress-related coping behaviors in adolescent mental health prevention and intervention. Strategies targeting socially evaluative academic pressures and adaptive emotion regulation may be beneficial in this population.

## Data Availability

The raw data supporting the conclusions of this article will be made available by the authors, without undue reservation.
