# Supplementary material for: Associations of academic stress with anxiety and depressive symptoms: the mediating role of emotional eating
Source: Front Nutr. 2026 May 4;13:1826577. doi: 10.3389/fnut.2026.1826577 (PMC13185491; doi:10.3389/fnut.2026.1826577)
Supplement: Supplementary file 1 [file Table_1.docx]

**Supplementary Table S1 Academic Stress Scale Items**

| **No.** | **Items (Response scoring: 1=Strongly Disagree; 2=Disagree; 3=Neutral; 4=Agree; 5=Strongly Agree)** |
| --- | --- |
| **1** | I often talk to my parents about what happens to me at school. |
| **2** | My parents often nag me about wanting me to be an outstanding person. |
| **3** | My parents blame me even for small mistakes. |
| **4** | My parents control my interests and hobbies. |
| **5** | The teacher criticizes me when I cannot answer their question. |
| **6** | I have a distant relationship with my teachers. |
| **7** | My parents get annoyed when I obtain poor exam results. |
| **8** | I believe further education is my only way out. |
| **9** | I feel sorry for my parents if I do not perform well in exams. |
| **10** | The teacher pays no attention to me and almost ignores my existence. |
| **11** | When I get home from school, the most frequent questions my parents ask are: “Have you had any exams lately? What’s your rank?” |
| **12** | I feel depressed if my exam results do not reach the excellent level. |
| **13** | I feel inferior when I am around classmates with good academic performance. |
| **14** | Even during holidays, I prefer staying alone in my room and rarely communicate with my parents. |
| **15** | My parents often criticize me in front of others, calling me lazy and unhardworking. |
| **16** | I am often dissatisfied with my own exam results. |
| **17** | I believe that only good academic grades can make others respect me. |
| **18** | The teacher tends to link negative incidents in the class to students with poor grades. |
| **19** | The teacher prefers to ask questions to classmates with good academic performance. |
| **20** | When I feel upset, I think I have no close friend to confide in. |
| **21** | I often have conflicts with my parents. |

The academic stress scale comprised four dimensions: parental pressure (6 items: 2, 3, 4, 7, 11, and 15), self-imposed pressure (6 items: 8, 9, 12, 13, 16, and 17), teacher pressure (5 items: 5, 6, 10, 18, and 19), and social pressure (4 items: 1, 14, and 20, and 21).

**Supplementary Table S2 Sensitivity analyses of the association between academic stress and anxiety/depressive symptoms**

| **Analysis Condition** | ***n*** | ***OR (95%CI)*** | |
| --- | --- | --- | --- |
|  |  | **Anxiety symptoms** | **Depressive symptoms** |
| **^a^ Limited population to:** |  |  |  |
| Good sleep quality | 561 | **1.07 (1.04-1.10)** | **1.07 (1.05-1.09)** |
| Residing in towns or cities | 465 | **1.07 (1.04-1.09)** | **1.06 (1.04-1.08)** |
| Good family economic | 365 | **1.07 (1.04-1.09)** | **1.06 (1.04-1.08)** |
| **^a^ Excluding individuals <10 years:** | 595 | **1.07 (1.05-1.09)** | **1.06 (1.04-1.08)** |
| **^a^ Excluding BMI >24** **kg/m^2^:** | 509 | **1.07 (1.04-1.09)** | **1.06 (1.04-1.07)** |
| **^b^ Inverse probability weighting:** | 608 | **1.08 (1.06-1.10)** | **1.07 (1.05-1.08)** |

^a^ Adjusted for sex (boy, girl), age (continuous), place of residence (city, town, rural), education (primary school, junior high school, senior high school, university), family economic status (good, poor), BMI (continuous), dietary diversity (continuous), sleep duration (continuous), sleep quality (good, poor), social media duration (continuous), sedentary duration (continuous), physical activity duration (continuous), body image (continuous).

^b^ Propensity weight was based on: sex (boy, girl), age (continuous), place of residence (city, town, rural), education (primary school, junior high school, senior high school, university), family economic status (good, poor), BMI (continuous), dietary diversity (continuous), sleep duration (continuous), sleep quality (good, poor), social media duration (continuous), sedentary duration (continuous), physical activity duration (continuous), body image (continuous).

Except for the factor itself. Bold values indicated statistical significance *P* <0.05.

**Supplementary Fig. S1 Restricted Cubic Spline for the association between dimensions of academic stress and anxiety symptoms**

| 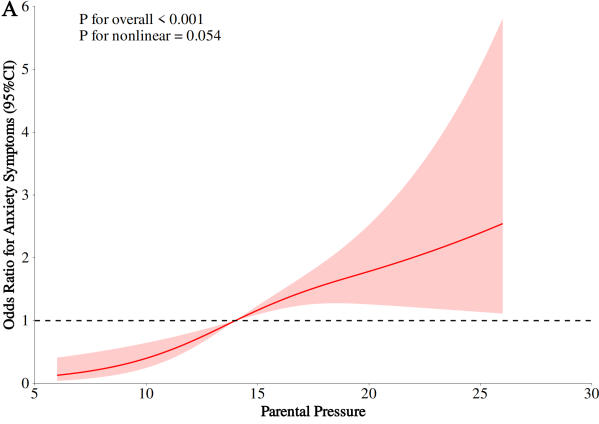 | 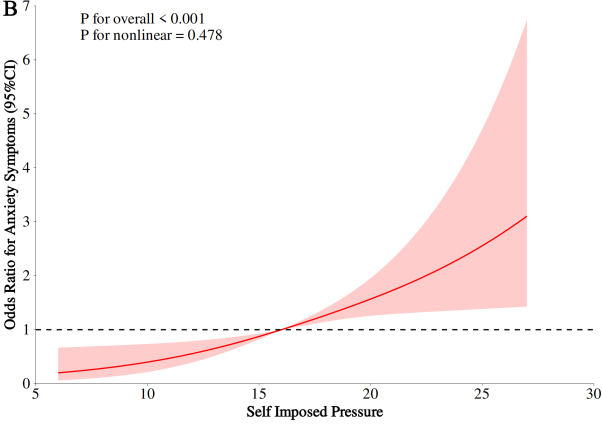 |
| --- | --- |
| 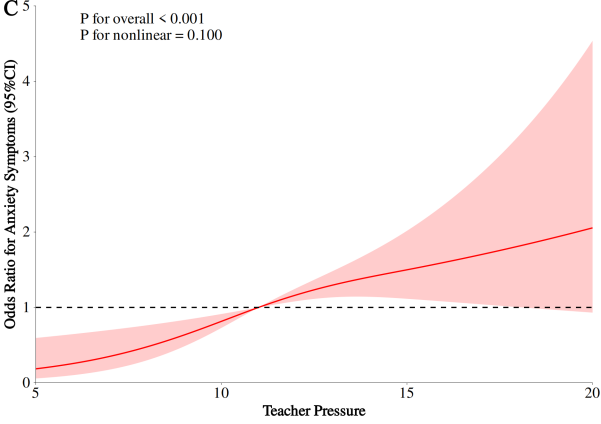 | 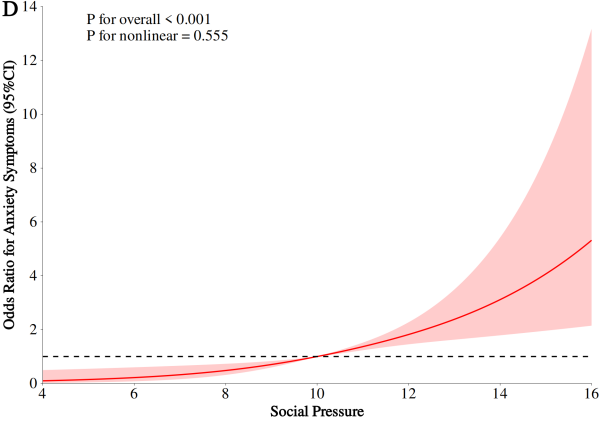 |

Note: (A) Parental pressure with anxiety symptoms, (B) Self-imposed pressure with anxiety symptoms, (C) Teacher pressure with anxiety symptoms, (D) Social pressure with anxiety symptoms.

Adjusted for sex (boy, girl), age (continuous), place of residence (city, town, rural), education (primary school, junior high school, senior high school, university), family economic status (good, poor), BMI (continuous), dietary diversity (continuous), sleep duration (continuous), sleep quality (good, poor), social media duration (continuous), sedentary duration (continuous), physical activity duration (continuous), body image (continuous).

**Supplementary Fig. S2** **Restricted Cubic Spline for the association between dimensions of academic stress and depressive symptoms**

| 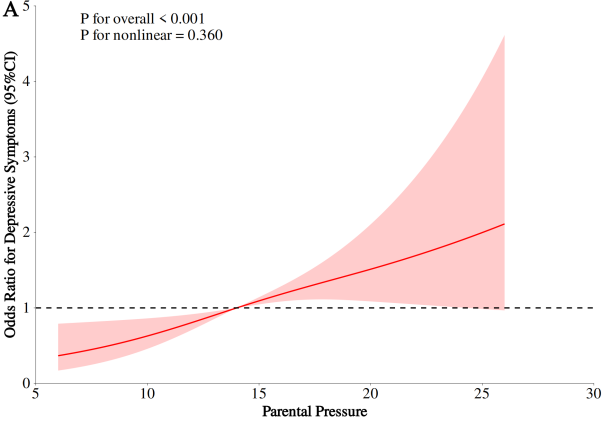 | 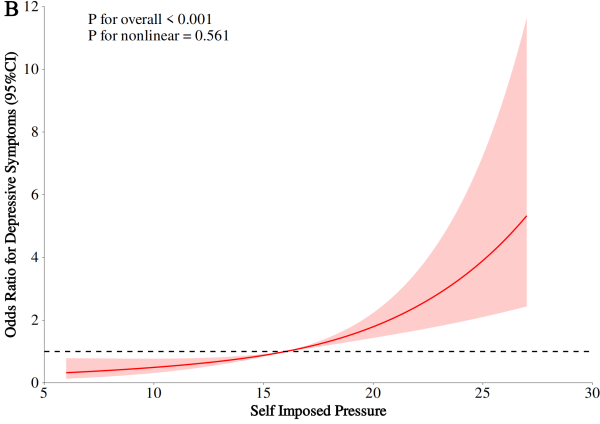 |
| --- | --- |
| 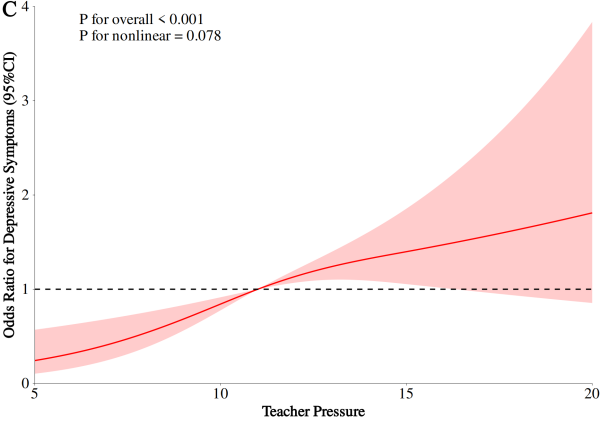 | 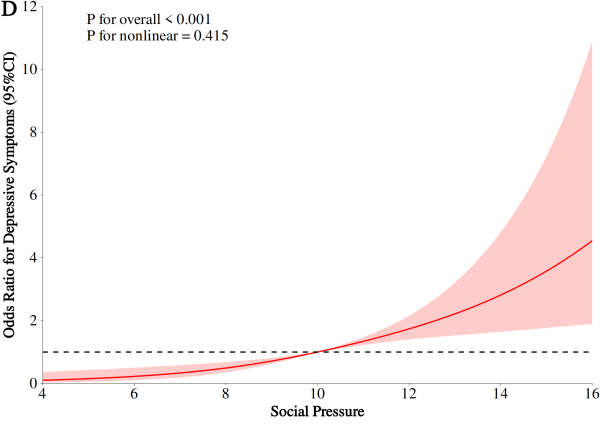 |

Note: (A) Parental pressure with depressive symptoms, (B) Self-imposed pressure with depressive symptoms, (C) Teacher pressure with depressive symptoms, (D) Social pressure with depressive symptoms.

Adjusted for sex (boy, girl), age (continuous), place of residence (city, town, rural), education (primary school, junior high school, senior high school, university), family economic status (good, poor), BMI (continuous), dietary diversity (continuous), sleep duration (continuous), sleep quality (good, poor), social media duration (continuous), sedentary duration (continuous), physical activity duration (continuous), body image (continuous).
